# Hepatitis B virus PreS2-mutant large surface antigen activates store-operated calcium entry and promotes chromosome instability

**DOI:** 10.18632/oncotarget.8109

**Published:** 2016-03-16

**Authors:** Tim Ting-Chung Yen, Anderson Yang, Wen-Tai Chiu, Tian-Neng Li, Lyu-Han Wang, Yi-Hsuan Wu, Hui-Chen Wang, Linyi Chen, Wen-Ching Wang, Wenya Huang, Chien-Wen Chang, Margaret Dah-Tsyr Chang, Meng-Ru Shen, Ih-Jen Su, Lily Hui-Ching Wang

**Affiliations:** ^1^ Institute of Molecular and Cellular Biology, National Tsing Hua University, Hsinchu 300, Taiwan; ^2^ Department of Biomedical Engineering, National Cheng Kung University, Tainan 701, Taiwan; ^3^ Center of Infectious Diseases and Signal Transduction, National Cheng Kung University, Tainan 701, Taiwan; ^4^ Institute of Pharmaceutics, Development Center for Biotechnology, Taipei 22180, Taiwan; ^5^ Institute of Molecular Medicine, National Tsing Hua University, Hsinchu 300, Taiwan; ^6^ Department of Medical Science, National Tsing Hua University, Hsinchu 300, Taiwan; ^7^ Department of Medical Laboratory Science and Biotechnology, National Cheng Kung University, Tainan 701, Taiwan; ^8^ Department of Biomedical Engineering and Environmental Sciences, National Tsing Hua University, Hsinchu 300, Taiwan; ^9^ Department of Pharmacology, National Cheng Kung University, Tainan 701, Taiwan; ^10^ National Institute of Infectious Diseases and Vaccinology, National Health Research Institutes, Tainan 704, Taiwan; ^11^ Department of Biotechnology, Southern Taiwan University of Science and Technology, Tainan 710, Taiwan

**Keywords:** SOCE, ER stress, hepatitis B virus, ground-glass hepatocytes, aneuploidy

## Abstract

Hepatitis B virus (HBV) is a driver of hepatocellular carcinoma, and two viral products, X and large surface antigen (LHBS), are viral oncoproteins. During chronic viral infection, immune-escape mutants on the preS2 region of LHBS (preS2-LHBS) are gain-of-function mutations that are linked to preneoplastic ground glass hepatocytes (GGHs) and early disease onset of hepatocellular carcinoma. Here, we show that preS2-LHBS provoked calcium release from the endoplasmic reticulum (ER) and triggered stored-operated calcium entry (SOCE). The activation of SOCE increased ER and plasma membrane (PM) connections, which was linked by ER- resident stromal interaction molecule-1 (STIM1) protein and PM-resident calcium release- activated calcium modulator 1 (Orai1). Persistent activation of SOCE induced centrosome overduplication, aberrant multipolar division, chromosome aneuploidy, anchorage-independent growth, and xenograft tumorigenesis in hepatocytes expressing preS2- LHBS. Chemical inhibitions of SOCE machinery and silencing of STIM1 significantly reduced centrosome numbers, multipolar division, and xenograft tumorigenesis induced by preS2-LHBS. These results provide the first mechanistic link between calcium homeostasis and chromosome instability in hepatocytes carrying preS2-LHBS. Therefore, persistent activation of SOCE represents a novel pathological mechanism in HBV-mediated hepatocarcinogenesis.

## INTRODUCTION

Hepatitis B virus (HBV) infection causes fulminant hepatitis and advanced liver disease. In endemic areas, most chronic liver diseases, including cirrhosis and hepatocellular carcinoma (HCC), are associated with HBV [1–3]. Two HBV-encoded oncoproteins have been identified, including X and large surface antigen (LHBS). The X protein functions as a robust gene transactivator and a binding partner of p53 [4]. In contrast, the role of LHBS in the development of HCC is not clear.

**Figure 1:**
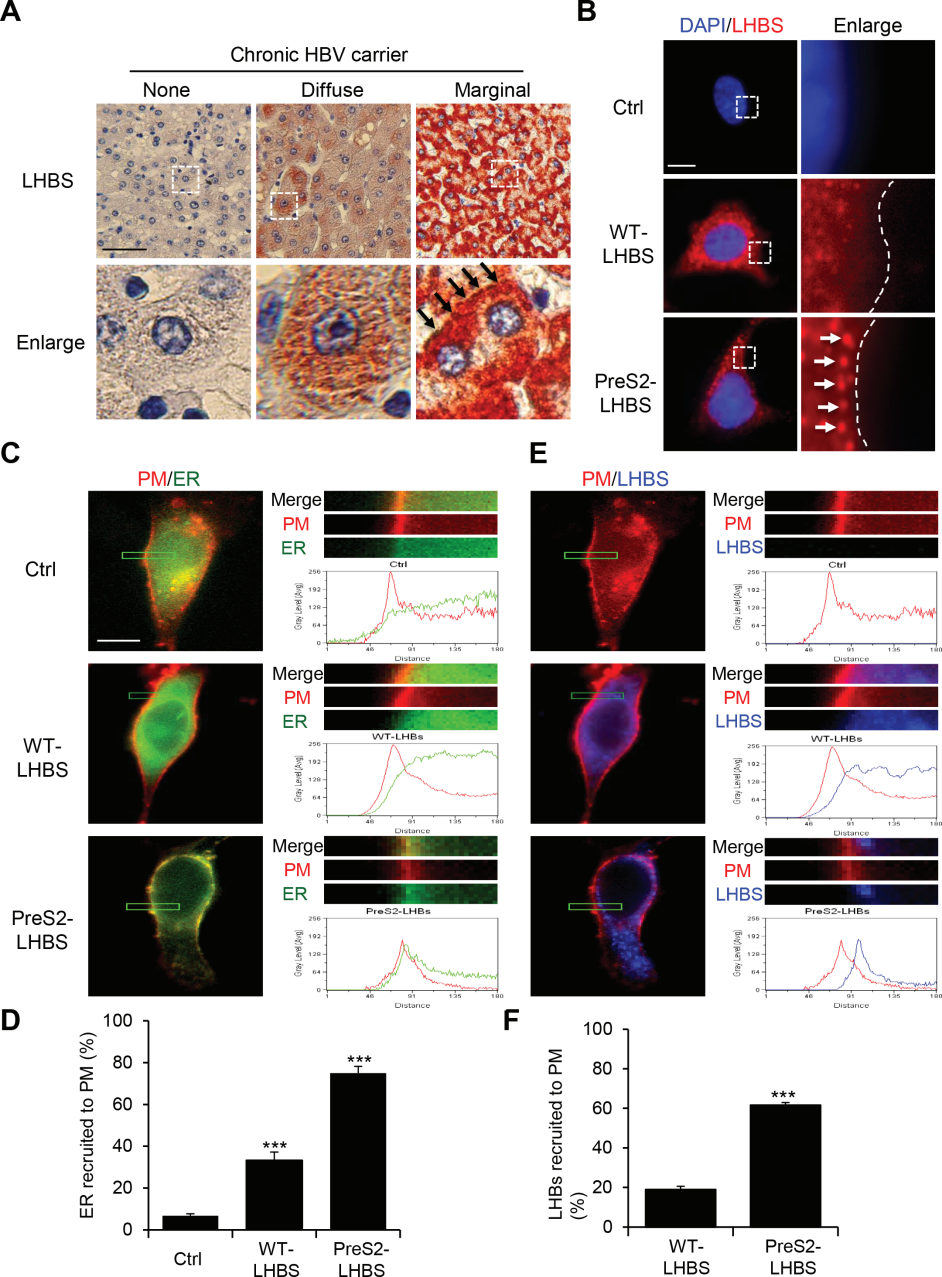
Intracellular distribution of the ER and LHBS (**A**) Immunohistochemical staining for LHBS was performed on cirrhotic liver sections obtained from chronic HBV carriers. Representative images displaying hepatocytes with absent, diffuse, and marginal LHBS expression patterns. Black arrows indicate peripheral distribution of LHBS in type II GGHs. Scale bar, 100 μm. (**B**) NeHepLxHT cells that expressed a control (Ctrl) tag, WT-LHBS, or preS2-LHBS were immunostained for LHBS (red) and counterstained with DAPI (blue). Enlarged images of the boxed areas are shown. Dashed lines indicate the edges of the cells. White arrows indicate the peripheral distribution of preS2-LHBS. Scale bar, 10 μm. (**C**, **E**) Immunofluorescence images of intracellular distributions of PM (red), ER (green), and LHBS (blue) are shown. The upper right panels show enlarged images of the boxed area from the corresponding left images. Line scanning profiles show the grey level signal intensity of the indicated markers in the enlarged area. Scale bar, 10 μm. (**D**) Quantitative results show that a percentage of cells displayed a peak ER signal intensity that overlapped with the PM, which indicates the peripheral ER. (**F**) Percentages of cells that displayed marginal LHBS are shown. Marginal recruitment of LHBS was defined by the detection of the peak signal intensity of LHBS that partially overlapped with the PM profile, as shown in the preS2-LHBS cells (**E**). ***p < 0.001.

During chronic HBV infection, viral surface antigen (HBsAg) is the most abundant viral product detected in the liver and circulation in patients. The surface antigen gene contains three segments (preS1, preS2, and S) with two internal translation start sites that produce three polypeptides with a common C-terminus: large (LHBS), middle (MHBS), and small surface antigens (HBS). Clinical assessment of HBsAg usually uses antibodies specific for the common S region to detect all three types of HBsAg. In early stages of chronic HBV infection, HBsAg exhibits diffused cytoplasmic distribution in the liver (Figure 1A). Occasionally, hepatocytes expressing inclusion-like HBsAg, known as type I ground glass hepatocytes (GGHs), can be found. In advanced disease, histological preneoplastic changes are detected in clusters of hepatocytes with a specific marginal distribution of HBsAg, known as type II GGHs or marginal type GGHs (Figure 1A) [5]. Notably, the development of different GGHs reflects the emergence of immune-escape mutants during chronic viral infection [6–9]. In addition, approximately 45% to 60% of HCC patients had preS mutants, suggesting these mutations are gain-of-function mutations during hepatocarcinogenesis [6, 10, 11]. In-frame truncations of the immune-dominant preS2 region with or without a preS2 start codon mutation were isolated from type II GGHs using laser capture microdissection and in the serum of patients with advanced liver diseases and HCC [7–9]. Strikingly, viral preS2 mutants were found in nearly half of children with HCC, in contrast to its absence in children with chronic HBV infection [11]. In another retrospective study of 42 children with HCC, preS deletion mutants were detected in over 90% of cases positive for HBV, among which 74% of cases contained preS2 deletion mutants [12]. Notably, the preS2 deletion consistently appeared with the deletion of nucleotides 4–57, corresponding to a loss of 18 amino acids, including one CD8 T cell epitope, in the livers or circulation [7, 9, 12, 13]. These studies implied that preS2 truncation mutants are immune-escape gain-of-function mutations that contribute to the development of HCC during chronic HBV infection.

The oncogenic characteristics of preS2 mutants have been investigated recently. Transgenic mice expressing the most typical preS2 mutant (nucleotides 4–57 deletion) LHBS protein developed liver dysplastic changes and ultimately HCC, indicating that preS2-LHBS is a promising viral oncoprotein [14, 15]. The retention of preS2-LHBS in the endoplasmic reticulum (ER) induced ER stress and thereby initiated the ER stress-dependent VEGF/Akt/mTOR and NF-κB/COX2 signaling pathways, as well as oxidative DNA damage and centrosome overduplication [16, 17]. Independent of the ER stress pathways, preS2-LHBS directly interacted with c-Jun activation domain-binding protein 1 (JAB1), which led to the degradation of p27 and thereby promoted the hyperphosphorylation of retinoblastoma [18]. In addition, preS2-LHBS directly interacted with importin α1, thereby blocked the nuclear transport of an essential DNA repair and recombinant factor, Nijmegen breakage syndrome 1 (NBS1), upon DNA damage, and subsequently impeded DNA double-stranded break repair [19]. Notably, transgenic mice carrying preS2-LHBS displayed higher genomic instability than transgenic mice carrying HBV X protein using array-based comparative genomic hybridization. Consistently, human type II GGHs carrying preS2 mutants also exhibited an increased level of DNA double-strand breaks [19]. These results indicate that HBV preS2 mutants may facilitate genomic instability in hepatocytes during disease progression. Notably, genomic instabilities were detected not only in individual genes but also at the chromosome level in these transgenic mice [19]. Similarly, the genetic compositions in HBV-associated HCC are complicated with changes in individual chromosomes, such as gains of chromosomes 1q/6p/8q and losses of chromosomes 4q, 8p, 16q, and others [20]. Although the induction of oxidative DNA damage and the inhibition of DNA repair could provide an explanation for the changes at the gene level, whether and how HBV promotes chromosome number instability during disease progression were not investigated.

In the present study, we aimed to explore whether the subcellular distribution of preS2-LHBS is essential for its oncogenic properties during disease progression. Unlike conventional ER-resident proteins that display typical perinuclear distribution in the cell, preS2-LHBS was mostly detected at the cell margin in type II GGHs and HuH-7 cells following transient transfection [9]. Marginal distribution of preS2-LHBS in GGHs prompted us to investigate whether this particular subcellular localization is involved in the ER-plasma membrane (PM) connection under stress conditions. In the present study, we show that the expression of preS2-LHBS increased ER and plasma membrane (PM) connections through the activation of store-operated calcium entry (SOCE), which is mediated by the interaction between stromal interaction molecule-1 (STIM1) and a calcium release-activated calcium modulator 1 (Orai1). The activation of SOCE not only increased the intracellular calcium concentration but also provoked centrosome overduplication. By time-lapse imaging, hepatocytes carrying preS2-LHBS underwent aberrant multipolar division and ultimately reached chromosome aneuploidy. Finally, we show that preS2-LHBS is capable of inducing xenograft tumorigenesis, and this effect was largely suppressed by the depletion of STIM1. Thus, we suggest that the activation of SOCE machinery is involved in chromosome instability in the development of HBV-mediated HCC.

## RESULTS

### PreS2-LHBS promotes ER-PM connections through ER stress

In contrast to the diffuse cytoplasmic distribution of wild type (WT)-LHBS, preS2-LHBS was usually detected at the cell margin of GGHs in the liver (Figure 1A). Similarly, WT-LHBS and preS2-LHBS displayed diffuse and marginal localization, respectively, in the hepatic progenitor line NeHepLxHT [21] (Figure 1B). As preS2-LHBS is an ER-resident protein, we asked whether the cytoplasmic distribution of bulk ER is affected in these cells. To this end, cells were infected with recombinant baculoviruses for exogenous expression of red fluorescent protein (RFP) fused with the myristoylation/palmitoylation sequence from Lck tyrosine kinase (as PM-RFP) and green fluorescence protein (GFP) fused with the ER signal sequence of calreticulin and KDEL (as ER-GFP). By monitoring the spatial correlation between ER-GFP and PM-RFP, we found that overlapping signals at the cell margin were increased in the presence of WT- and preS2-LHBS (Figure 1C). Quantitatively, the overlapping peak signals associated with ER-GFP and PM-RFP were detected in approximately 30% of the WT-LHBS cells and 75% of the preS2-LHBS cells. Less than 5% of the control cells displayed marginal ER-GFP (Figure 1D). Coordinately, greater than 60% of the preS2-LHBS cells displayed peripheral recruitment of preS2-LHBS, as indicated by the peak fluorescence signal shown near PM-RFP (Figure 1E, 1F). Thus, we concluded that the ER-PM connections were increased in the presence of preS2-LHBS.

**Figure 2:**
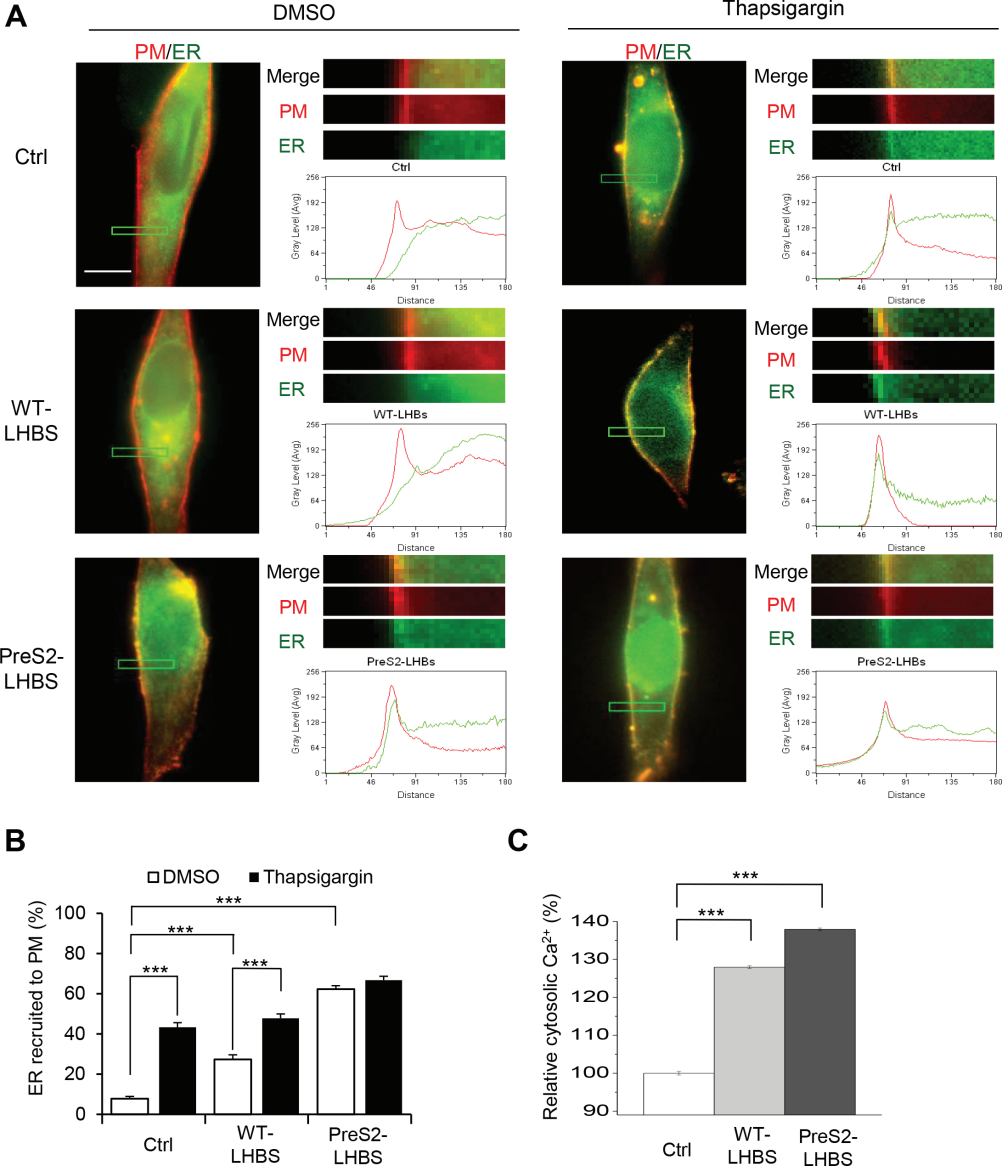
ER stress increases ER-PM connections (**A**) Subcellular localization of PM (red) and ER (green) in indicated stable cell lines following the induction of ER stress. Cells were pre-treated with BacMam baculoviruses for the transduction of ER-GFP and PM-RFP for 48 h, followed by a 15-min treatment with DMSO or thapsigargin. The upper right panels show enlarged images of the boxed area from the corresponding images. Line scanning profiles show the grey level signal intensity of ER (green line) and PM (red line) in the enlarged area. Scale bar, 10 μm. (**B**) Percentage of cells with ER recruitment toward the PM is defined by the detection of the ER peak signal intensity that overlapped with the PM peak profile. (**C**) The intracellular calcium concentration was measured by Fura-2 intensity. ***p < 0.001.

To investigate whether ER stress is involved in the ER-PM connection, we treated cells with thapsigargin, which inhibits SERCA and activates ER stress via the depletion of ER calcium storage. Peripheral localization of ER-GFP was significantly increased in control and WT-LHBS cells with thapsigargin. In contrast, the marginal distribution of ER-GFP was not affected by thapsigargin in preS2-LHBS cells (Figure 2A, 2B). Given that ER stress is expected to increase calcium efflux from the ER, we confirmed that the preS2-LHBS cells displayed increased intracellular calcium levels compared with the control and WT-LHBS cells (Figure 2C). Together, these results indicated that preS2-LHBS promotes the ER-PM connection and disrupt calcium homeostasis through ER stress.

### SOCE provokes the marginal distribution of LHBS

**Figure 3:**
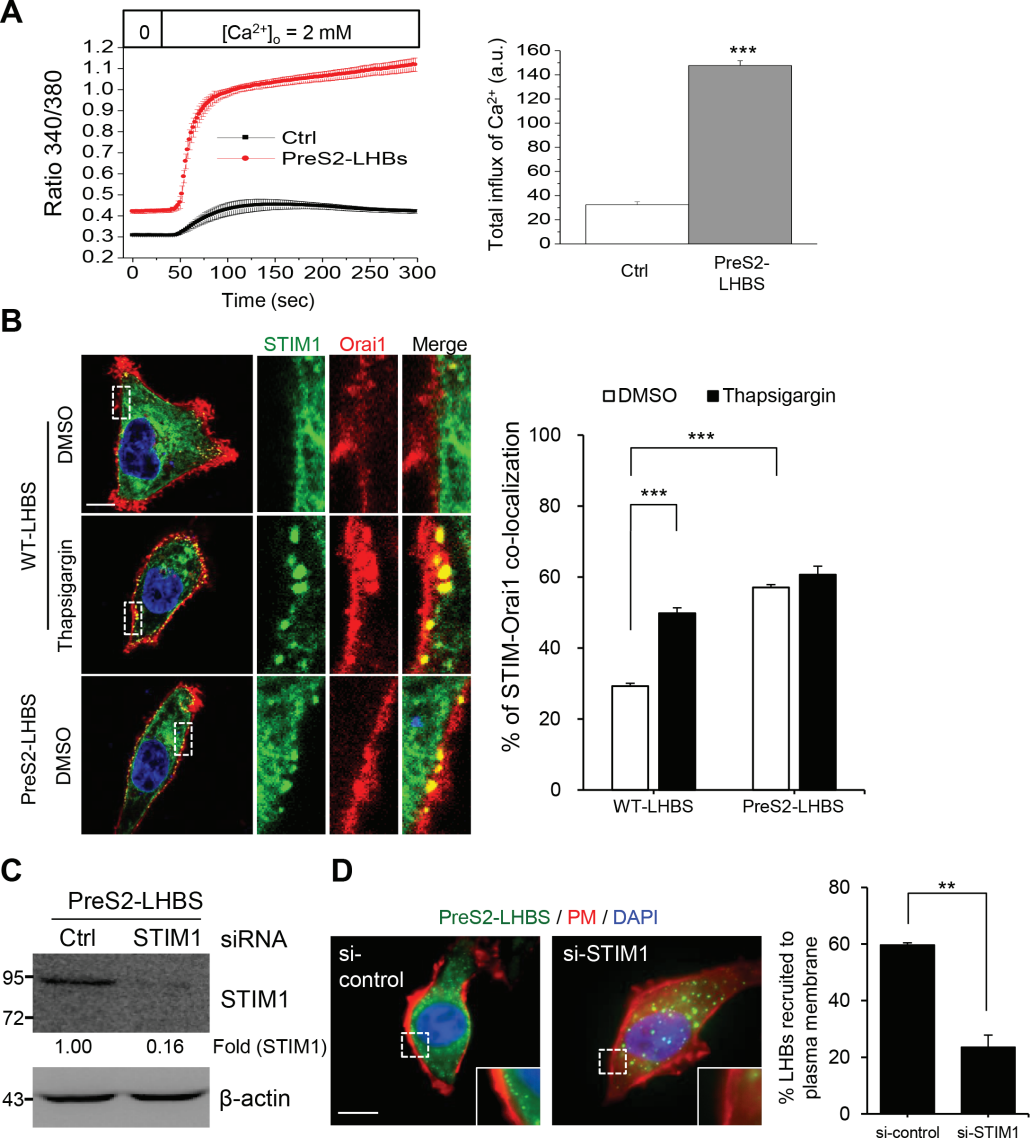
SOCE activation and peripheral recruitment of STIM1 in hepatocytes under ER stress (**A**) SOCE activity was examined in control and preS2-LHBS cells by measuring calcium influx over time after being released from calcium-free medium and thapsigargin. The quantitative results obtained from at least 50 cells are displayed in the right panel (a.u., arbitrary units). (**B**) STIM1 (green) and Orai1 (red) formed puncta at the cell margin in preS2-LHBS cells. In WT-LHBS cells, STIM1-Orai1 puncta were only detected after thapsigargin treatment. The percentage of cells that contained overlapping STIM1-Orai1 puncta at the cell periphery is shown in the right panel. Scale bar, 10 μm. (**C**) STIM1 depletion in preS2-LHBS cells was confirmed by Western blotting. The numbers below the blot indicate the STIM1 fold reduction. (**D**) Depletion of STIM1 abolished the recruitment of preS2-LHBS toward the cell margin. Quantitative results are presented in the right panel. **p < 0.01; ***p < 0.001.

Store-operated calcium entry is a major calcium influx pathway controlling the intracellular calcium concentration in the cell [22]. To investigate SOCE activity, cells were pre-loaded with a fluorescent calcium sensor, treated with thapsigargin, and incubated in calcium-free medium to deplete all intracellular calcium. SOCE activity was then measured via the detection of calcium influx after thapsigargin was removed and replaced with calcium-containing medium. Compared with the control cells, a significant increase in total calcium influx was detected in the preS2-LHBS cells (Figure 3A). Given that SOCE-mediated calcium influx depends on the interaction between STIM1 and Orai1 [23], the formation of STIM1-Orai1 puncta was detected in preS2-LHBS cells. In WT-LHBS cells, the STIM-Orai1 puncta were only detected after thapsigargin treatment (Figure 3B). Next, we explored whether the marginal distribution of preS2-LHBS depended on the peripheral recruitment of STIM1. Notably, the depletion of STIM1 abolished the peripheral recruitment of preS2-LHBs (Figure 3C, 3D). These results indicated that the marginal distribution of preS2-LHBS is mediated through the recruitment of STIM1 toward PM-localized Orai1 following the activation of SOCE machinery.

### Persistent SOCE activation depends on calcium efflux from the ER

**Figure 4:**
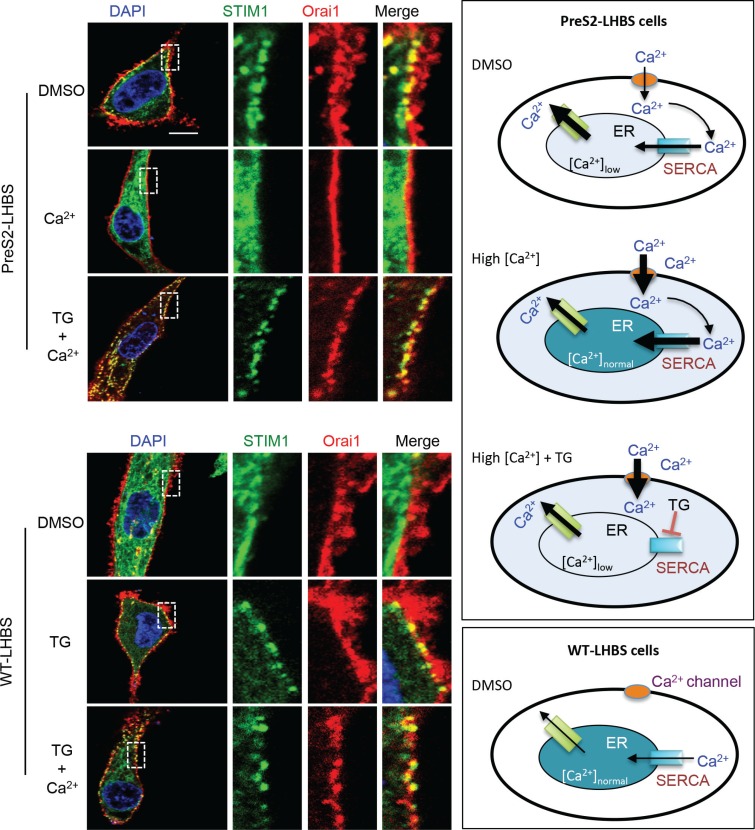
Activation of SOCE depends on the calcium concentration in the ER EGFP-STIM1 (green) and mOrange-Orai1 (red) were transiently expressed in WT-LHBS and preS2-LHBS following treatment with DMSO, thapsigargin (TG), and/or an additional 4 mM Ca^2+^ in culture media. Representative images are shown on the left. Right panels illustrate the relative concentration of cytosolic and ER calcium under various conditions. In preS2-LHBS cells, ER stress promotes calcium release from the ER and thereby induces the STIM1-Orai1 connection and SOCE activation. Replenishment of ER calcium via an increase in the calcium concentration in the culture medium abolished the STIM1 and Orai1 connection in preS2-LHBS cells. In contrast, co-treatment with TG restored SOCE activation in preS2-LHBS cells as a result of the blockage of SERCA-dependent calcium entry into the ER. These results indicate that a persistent STIM1-Orai1 connection depends on the calcium concentration in the ER. Scale bar, 10 μm.

Type II GGHs with marginally localized LHBS were persistent in the liver in advanced stages of chronic HBV infection [5, 6, 24, 25]. These observations implied that ER-PM connections are maintained constantly in type II GGHs. Given that the STIM1-Orai1 interaction is critical for this ER-PM connection, we asked whether the restoration of ER calcium homeostasis may retract the ER-PM connection in preS2-LHBS cells. To reduce ER calcium leakage in these cells, we increased the cytoplasmic calcium by adding extra calcium in the culture medium (final calcium concentration of 5 mM). Strikingly, STIM1-Orai1 puncta in preS2-LHBS cells were lost after adding extra calcium (Figure 4). Further treatment with thapsigargin, which blocked the ER calcium influx, rescued the formation of STIM1-Orai1 puncta in preS2-LHBS cells under a high calcium condition (Figure 4, upper panels). Similarly, we showed that thapsigargin induced STIM1-Orai1 puncta in WT-LHBs cells, which persisted in the high calcium condition (Figure 4, lower panels). Thus, insufficient calcium storage in the ER is likely the major driving force for the connection between STIM1 and Orai1. These results implied that persistent STIM1-Orai1 connections in preS2-LHBS-expressing cells depend on constant calcium efflux from the ER, as a result of persistent ER stress.

### SOCE promotes centrosome overduplication, multipolar division, and aneuploidy

**Figure 5:**
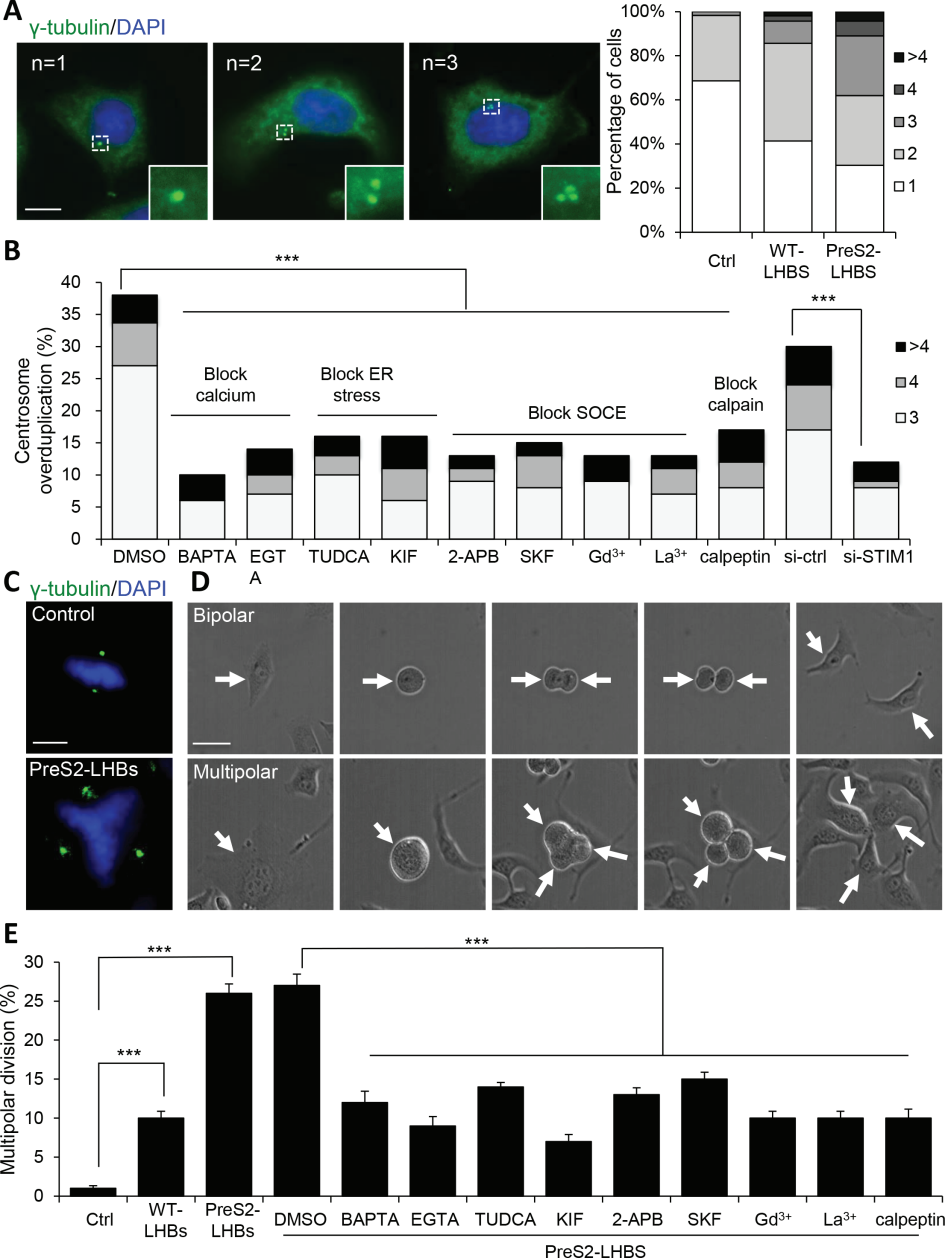
SOCE promotes centrosome overduplication and multipolar division (**A**) Representative immunofluorescence images of cells carrying 1 to 3 centrosomes, as indicated by **γ**-tubulin (green)-positive foci. Scale bar, 10 μm. Overall population of cells carrying different number of centrosomes in control, WT-LHBS, and preS2-LHBS cells is shown in the right panel. At least 200 cells were counted in each group, and the percentages of cells carrying different number of chromosomes are shown. (**B**) Centrosome overduplication in preS2-LHBS cells was suppressed by calcium chelators (BAPTA-AM, EGTA) and inhibitors of ER stress (TUDCA, KIF), SOCE (2-APB, SKF96365, Gd^3+^, La^3+^), and calpain (calpeptin), as well as by the depletion of STIM1. Percentage of cells carrying 3 or more centrosomes following these treatments are shown. (**C**) Representative images showing cells that underwent bipolar (upper) or multipolar (lower) chromosome alignment during mitosis. Spindle poles and chromosomes are indicated by **γ**-tubulin (green) and DAPI (blue), respectively. Scale bar, 10 μm. (**D**) Time-lapse DIC images displaying cells undergoing bipolar and multipolar divisions. Arrows indicate either the mother or daughter cells as appropriate. Scale bar, 10 μm. (**E**) Percentages of cells that underwent multipolar division are shown as indicated. Multipolar division in preS2-LHBS cells was suppressed upon inhibition of ER stress, SOCE, or calpain. ***p < 0.001.

A potential outcome of constant SOCE activation is the activation of calcium-dependent calpain proteases because of elevated intracellular calcium. Notably, calpain-dependent cyclin A proteolysis has been implicated in centrosome overduplication in HBV-mediated hepatocarcinogenesis [26]. We measured the number of centrosomes in hepatocytes with or without LHBS. Compared with the control cells, centrosome overduplication (centrosome number ≥ 3) was increased by 8.6− and 22.8-fold in WT-LHBS and preS2-LHBS cells, respectively (Figure 5A). The inhibition of ER stress, SOCE, and calpain largely suppressed centrosome overduplication in preS2-LHBS cells. Specifically, a bile acid derivative, tauroursodeoxycholic acid (TUDCA), was used as a chemical chaperone to reduce ER stress [26]. Kifunensine (KIF) was used to reduce ER stress by suppressing ER-associated protein degradation [27]. As expected, the STIM1-depleted cells also displayed reduced centrosome numbers (Figure 5B). Using γ-tubulin and chromatin staining, we found that preS2-LHBS cells displayed abnormal chromosome alignment and multiple spindle poles during mitotic progression (Figure 5C). To further explore the consequence of centrosome overduplication, we monitored mitotic progression using time-lapse imaging and found that approximately 10% of the WT-LHBS and 26% of the preS2-LHBS cells underwent aberrant multipolar division (Figure 5D and Supplementary Movies). Furthermore, the inhibition of ER stress, SOCE, and calpain, as well as treatment with calcium chelators, reduced aberrant multipolar division in preS2-LHBS cells (Figure 5E).

**Figure 6:**
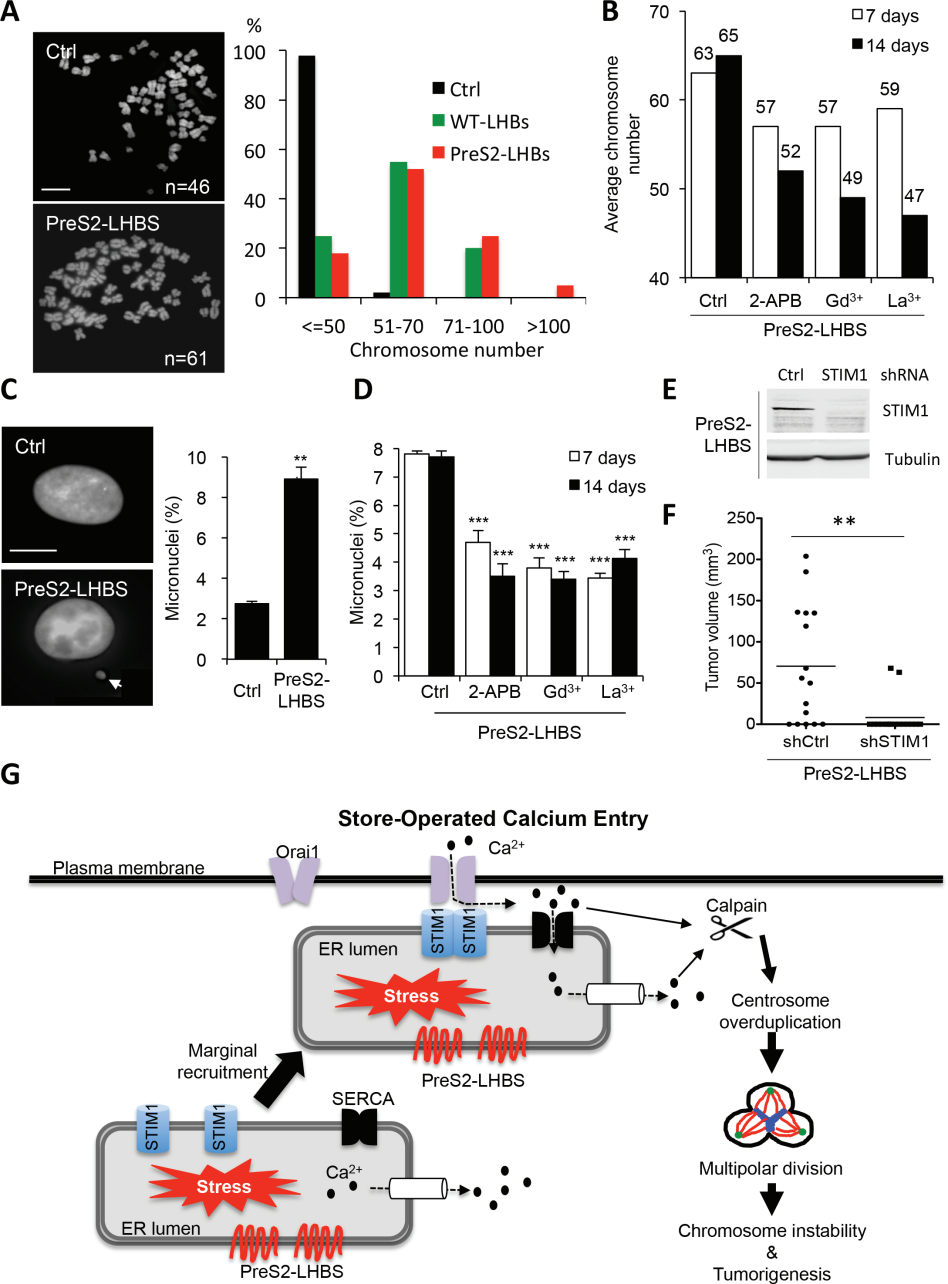
PreS2-LHBS promotes SOCE-dependent chromosome instability (**A**) Overall chromosome numbers of control and LHBS-positive cells were examined by chromosome spreading. Representative chromosome spreads in control and preS2-LHBS cells are shown on the left. Scale bar, 10 μm. At least 200 spreads were counted per experiment. Quantitative chromosome number distribution is shown on the right. (**B**) The average chromosome number in preS2-LHBS cells was reduced following long-term SOCE inhibition by 2-APB, Gd^3+^, and La^3+^. At least 200 spreads were counted per experiment. Numbers shown on top of each column indicate the average chromosome numbers. (**C**) An increased number of cells that carried micronuclei (arrow-indicated) was detected in preS2-LHBS cells stained with DAPI. Scale bar, 10 μm. (**D**) The percentage of preS2-LHBS cells that contained micronuclei was reduced following long-term SOCE inhibition. ***p < 0.001. (**E**) Depletion of STIM1 protein expression in preS2-LHBS cells was confirmed upon treatment of recombinant lentiviruses carrying STIM1 shRNA. Tubulin expression was detected as an internal control. (**F**) STIM1 knockdown suppressed xenografted tumorigenesis of preS2-LHBS cells. PreS2-LHBS cells infected with recombinant lentiviruses for the expressions of control or STIM1 shRNA were injected subcutaneously into nude mice for the observation of *in vivo* tumorigenesis for 2 months. Tumor volumes are presented as scatter dot plot with mean values (*n* = 16). **p < 0.01. (**G**) Illustration of the cause and consequences of persistent ER-PM connections in type II GGHs. PreS2-LHBS triggers ER stress and promotes calcium release from the ER. Reduced ER calcium storage provokes STIM1 oligomerization and the recruitment of the STIM1-resident ER toward the PM-localized Orai1. These actions initiate calcium influx, increase cytoplasmic calcium concentrations, and thereby provoke calpain activation and centrosome overduplication. Mitosis that proceeds with extra centrosomes results in abnormal multipolar division and ultimately produces chromosome instability.

As a direct outcome of aberrant multipolar division is chromosome number instability, we continued to investigate changes in overall chromosome numbers in hepatocytes in the presence of preS2-LHBS. The parental line NeHepLxHT used in this study is diploid, and more than 95% of cells contain 46 chromosomes [21]. This chromosome stability was maintained in the established control line but was disrupted in cells carrying WT-LHBS and preS2-LHBS (Figure 6A). The average chromosome numbers in WT-LHBS and preS2-LHBS cells were 60 and 65, respectively. Whereas the majority of WT-LHBS and preS2-LHBS cells had 51–70 chromosomes, cells carrying more than 100 chromosomes were detected only in preS2-LHBS cells (Figure 6A). Thus, preS2-LHBS has a stronger impact on chromosome instability than WT-LHBS cells. Next, we investigated whether the inhibition of SOCE machinery may block preS2-LHBS-induced chromosome instability. To this end, preS2-LHBS cells were treated with SOCE inhibitors for 7 and 14 days before harvesting for chromosome karyotyping. As expected, long-term inhibition of the SOCE machinery by 2-APB, Gd^3+^, or La^3+^ significantly reduced chromosome instability in preS2-LHBS cells in two weeks (Figure 6B and Supplementary Figure S1). Apart from chromosome instability, we also detected a 3-fold increase in the formation of micronuclei in preS2-LHBS cells. Coordinately, micronucleation in preS2-LHBS cells was decreased by long-term SOCE inhibition (Figure 6C, 6D). Finally, we investigated whether SOCE is essential for preS2-LHBS-mediated tumorigenesis in immune-deficient mice. PreS2-LHBS cells were infected with recombinant lentiviruses that expressed control or STIM1 shRNA. Successful depletion of STIM1 upon lentivirus treatment was confirmed by western blotting (Figure 6E). Next, a total of 1 × 10^6^ lentivirus-treated cells were injected subcutaneously into nude mice for the observation of xenograft tumorigenesis. Tumorigenesis in the control and shSTIM1 groups occurred in 69% (11/16) and 13% (2/16) of mice, respectively. In addition, tumor volume was significantly reduced in the shSTIM1 group (Figure 6F). Together, these results implied that the activation of SOCE machinery contributed to centrosome overduplication, chromosome instability, and *in vivo* tumorigenesis in hepatocytes carrying preS2-LHBS (Figure 6G).

## DISCUSSION

Since their initial discovery in the early 1970s, GGHs have been implicated as a diagnostic biomarker of chronic HBV infection and as a preneoplastic lesion for the development of HCC [6, 25, 28]. Here we provide a mechanistic link between ER stress, SOCE, and chromosome instability in hepatocytes carrying preS2-LHBS, a leading cause of type II GGHs. We show that ER stress increased the ER-PM connection through the STIM1-Orai1 interaction, which subsequently initiated SOCE and thereby contributed to centrosome overduplication, multipolar division, and chromosome instability.

ER stress is a self-defense mechanism that is triggered by the presence of unfolded/malfolded proteins (also known as the unfolded protein response, UPR), the accumulation of these proteins (ER-overloaded response, EOR), or the disruption of calcium homeostasis in the ER [29, 30]. During chronic HBV infection, mutations occurred in the preS regions were found in GGHs with the accumulation of surface antigens, indicating that these proteins are trapped in the ER due to their inability to undergo proper folding [9]. PreS2-LHBS triggers both UPR and EOR, as implied from activations of UPR and EOR downstream signaling pathways [6, 14, 26, 31–33]. As a secondary effect, the stress also leads to a sustained calcium leak from the ER and thereby increases the cytosolic calcium concentration [34]. This calcium effect is likely mediated by the calcium release channels ryanodine-receptor (RyR) or inositol 1,4,5-trisphosphate (IP_3_)-receptor (IP_3_R) or the newly identified translocon [35, 36]. As a result, calcium is reduced in the ER, which can be rescued by increasing the calcium influx from extracellular resources, thereby replenishing ER calcium [37]. This mechanism is named store-operated calcium entry/SOCE because the calcium influx is triggered by calcium storage in the ER [38].

STIM1 is a calcium sensor in the ER. It has a single transmembrane domain with a calcium-binding EF-hand motif near the N-terminus, which is located in the ER lumen [39]. A decrease in ER luminal calcium concentration results in the dissociation of calcium from the EF-hand motif, which, in turn, leads to STIM1 oligomerization [40]. Accordingly, ER stress-induced calcium leak provoke STIM1 oligomerization. A recent model predicted that STIM1 dimers are involved in crosslinking Orai1 channels, with implications for the kinetics and localization of Orai1 channel opening [41]. Thus, the formation of STIM1-Orai1 clusters indicates not only the ER-PM junction sites but also where calcium enters the cell.

The present study implies that preS2-LHBS is recruited toward the plasma membrane, along with other ER resident proteins, upon SOCE activation. To support this notion, increased peripheral distribution of ER-resident GRP78 was detected in preS2-LHBS cells and in control lines after thapsigargin stimulation (Supplementary Figure S2). Based on the following reasons, we conclude that the marginal recruitment of preS2-LHBS depends on interactions between STIM1 and Orai1 but not LHBS. First, STIM1 depletion is sufficient to abolish the recruitment of preS2-LHBS to the cell margin (Figure 3C). Second, preS2-LHBS is not directly associated with STIM1 (Supplementary Figure S3). Third, increased ER-PM connection was detected upon SOCE activation in control cells even in the absence of LHBS (Figure 2A). These results imply that the marginal distribution of preS2-LHBS is a result of ER stress-mediated SOCE activation, not by the protein-protein interaction between preS2-LHBS and STIM1.

What is unique for the induction of SOCE during chronic HBV infection? Once the ER calcium concentration is replenished, SOCE should be inactivated, and the ER returns to the resting stage and dissociates from the PM. However, marginal distribution of preS2-LHBS is constantly detected in type II GGHs (Figure 1A), indicating that the stress is never resolved in these hepatocytes. What keeps SOCE active in type II GGHs is the next question to be solved. We reasonably suspect that ER calcium influx may quickly leak out again in the presence of preS2-LHBS. To support this notion, preS2-LHBS was treated with the high calcium condition in which the ER calcium leak is prevented by high cytosolic calcium concentration. In this condition, STIM1-Orai1 connected puncta were dissociated in preS2-LHBS cells, indicating that the persistent STIM1-Orai1 interaction depends on ER calcium storage (Figure 4). Furthermore, high calcium environments did not disrupt STIM1-Orai1 interactions when ER calcium replenishment was blocked by thapsigargin. These results imply that persistent ER stress, which constantly promotes the efflux of calcium from the ER, sustains SOCE activation in type II GGHs. As the stress conditions were not resolved, the interaction between STIM1 and Orai1 persisted in the cells.

PreS truncations are known as gain-of-function oncogenic mutations. PreS2-LHBS may promote hepatocarcinogenesis through ER stress-dependent and -independent pathways [17, 42]. Notably, ER stress alone is insufficient to induce carcinogenesis because cell cycle progression may be attenuated by downstream signaling [30]. Alternatively, hepatocytes may cope with ER stress via an adaptive UPR, which includes the enhancement of protein folding and degradation in the ER [43]. This is likely the case for GGHs because ER chaperone proteins are increased in GGHs that carry preS1 and preS2 mutants in the liver [44]. In addition, preS2-LHBS may act as a gene transactivator to activate PKC/c-Raf-1/MAP2 kinase, NF-κB, and mTOR signal cascades, as well as cyclin A and Bcl-2 upregulation, to facilitate cell proliferation [14, 45–48]. The present study implies that type II GGHs are prone to aneuploidy if mitotic division occurs in the presence of extra centrosomes. Exactly how much genetic information was changed in type II GGHs is not clear. Notably, hepatocytes of various ploidy classes (4 n, 8 n, and 16 n) typically emerge during liver postnatal development [49]. It will be of interest to explore the influence of HBV on genomic integrity in the context of diverse hepatocyte polyploidy in the future.

Still, why it takes decades to develop HCC in patients with chronic HBV infection is unknown. We consider two possible explanations. First, during the early stages of HBV infection, hepatocytes expressing viral antigens are obviously eliminated by host immunity, and therefore, the expansion of virus-infected hepatocytes is likely limited. This immune pressure pushes the emergence of preS mutants, as a result of immune escape. This is supported by the increasing prevalence of type II GGHs and preS2 mutants in advanced stages of chronic HBV infection [6]. Second, HCC is characterized with complex genomic composition [20, 50, 51], implying that the development of HCC requires the accumulation of multiple genetic changes during disease progression. Whereas the development of preS2 mutants facilitates genetic defects, endogenous tumor suppressor genes can still limit aberrant cell cycle progression of virus-infected hepatocytes until their functionalities are compromised by further genetic changes. Thus, the risk of HCC was increased by time in chronic HBV carriers [52]. If the effect of host immunity is removed, the presence of a preS2 mutant alone should speed up the disease progression. In fact, the presence of preS mutants is a known risk of liver cirrhosis and HCC [53]. In addition, preS2 mutants were detected in nearly half of all children with HCC [11]. Accordingly, whether and when immune-escape and oncogenic preS2 mutants occur in the liver may set the clock for advanced disease progression in patients with chronic HBV infection.

Apart from preS2-LHBS, another viral oncoproduct, HBx, was shown to affect calcium signaling in hepatocytes through a completely different mechanism. HBx interacts directly with Bcl-2 and Bcl-xL through its Bcl-2-homology-3-like motif, and this interaction not only elevates cytosolic calcium but also provokes cell death and viral replication [54]. The inhibition of mitochondrial calcium channels blocked HBx-mediated HBV replication, indicating that cytosolic calcium is regulated by limited mitochondrial calcium uptake [55]. Unlike preS2-LHBS, which can be abundantly detected in type II GGHs, the expression of HBx is often limited in HCC and a small population of surrounding parenchyma cells [56]. In cirrhotic livers, HBx was detected in small subpopulations of type II GGHs [57]. The co-expression of HBx and preS2-LHBS displayed a synergistic effect on several oncogenic signals, such as vascular endothelial growth factor-A, Akt, and mTOR, as well as xenograft tumorigenesis [57]. Whether and how HBx coordinates with preS2-LHBS in the development of HBV-related HCC requires further investigation.

In conclusion, the activation of SOCE machinery explains the marginal recruitment of preS2-LHBS and the subsequent chromosome instability. To the best of our knowledge, this study provides the first mechanistic link between calcium homeostasis and chromosome instability in the pathogenesis of HBV. Notably, SOCE was shown to be essential for the migration, invasion, and proliferation of hepatoma cells and cervical carcinoma [58, 59]. Together, these studies highlight SOCE as a promising therapeutic target to control disease progression during chronic HBV infection.

## MATERIALS AND METHODS

### Cell culture, treatments, and bacmam transduction

The immortalized hepatic progenitor cell line NeHepLxHT [21] was purchased from and cultured as suggested by American Type Culture Collection (ATCC, Manassas, VA, USA). To generate stable cell lines, NeHepLxHT cells were infected with recombinant lentiviruses that carried SNAP (as a control) or SNAP-tagged WT and preS2 mutant LHBS genes (deletion nucleotides 2–55) and selected by 2 μg/ml puromycin. Viable cells were transiently labeled with cell-permeable SNAP-505 dye (New England Biolabs, MA, USA) and subjected to cell sorting. The following reagents were used: TG (thapsigargin, 2 μM, Sigma-Aldrich, St Louis, MO, USA), KIF (kifunensine, 50 μM, Sigma-Aldrich), TUDCA (tauroursodeoxycholic acid [sodium salt], 100 μM, Sigma-Aldrich), EGTA (ethylene glycol tetraacetic acid, 100 μM, Biokit, Miaoli, Taiwan), BAPTA-AM (4 μM, Calbiochem, San Diego, CA, USA), and calpeptin (20 μM, Calbiochem). The SOCE inhibitors (2-APB, SKF96365, Gd^3+^, and La^3+^) and siRNA targeting STIM1 were used as previously described [60]. STIM1 and Orai1 subcellular localization was monitored via transient transfection of cells with plasmids that encoded mOrange-Orai1 and EGFP-STIM1 for 48 hr. To monitor ER and PM localization, the cells were treated with BacMam baculovirus (CellLight ER-GFP and PM-RFP, Molecular Probes, Eugene, OR, USA) for 48 hr prior to fixation.

### Immunohistochemical and double immunofluorescence staining

For hepatic LHBS staining, paraffin-embedded tissues were pre-warmed at 65°C for 15 min and treated with xylene for paraffin removal. Antigen retrieval was performed by boiling the samples in a tissue pretreatment buffer solution (Invitrogen) for 30 min. The slides were then incubated with mouse anti-preS1 for 1 hr and assessed using the EnVision+ System-HRP kit (DAKO, Carpintena, CA, USA). For double immunofluorescence staining, the cells were fixed and stained with the indicated primary antibodies for 1 hr, followed by secondary antibodies conjugated with Alexa-488, Alexa-594, or Alexa-647 (Molecular Probes). The cells were mounted with mounting medium that contained DAPI. The images were acquired with a Zeiss LSM780 confocal microscope or an automated Leica DMI6000 inverted microscope equipped with an HCX PL FL 20x/NA0.4 objective and an EMCCD camera (Andor Luca R, Belfast, UK) as indicated. The following primary antibodies were used in this study: mouse anti-preS1 (a gift from Ningshao Xia, Xiamen University, China), rabbit anti-SNAP tag (P9310S, New England Biolabs, MA, USA), rabbit anti-STIM1 (PA5-23623, Thermo Scientific, IL, USA), and mouse anti-γ-tubulin (sc-17787, Santa Cruz, Dallas, Texas, USA).

### Monitoring mitotic progression by time-lapse live cell microscopy

For live cell imaging, individual cells were isolated and cultured in 35-mm Petri dishes (Ibidi, Germany) for a minimum of 12 hr prior to imaging. The cells were maintained on a microscope stage incubator at 37°C with a humidified atmosphere and 5% CO_2_ throughout the experiment. Multi-positional time-lapse imaging was performed using an automated Leica DMI6000 inverted microscope equipped with an HCX PL FL 20x/NA0.4 objective and an Andor Luca R EMCCD camera. To monitor mitotic cell progression, sequential differential interference contrast (DIC) images of the cells were obtained at 10-min intervals for 24 hr and analyzed using MetaMorph software. At least 100 cells were monitored per experiment, and the experiment was independently repeated three times.

### Measuring intracellular calcium and SOCE activity

The total cellular calcium concentration was measured by loading cells with Fura-2/acetoxymethyl ester (Fura-2/AM, Molecular Probes) at 37°C for 30 min. The cells were washed three times with phosphate-buffered saline (PBS) and imaged. To monitor calcium influx and SOCE activity, the cells were loaded with Fura-2/AM at 37°C for 30 min and subsequently incubated in a calcium-free medium that contained 2 μM thapsigargin for 30 min to deplete intracellular calcium stores. Calcium influx was measured after the addition of 2 mM calcium to the culture medium. The cells were imaged using an Olympus IX71 inverted microscope equipped with a xenon illumination system, an IMAGO CCD camera, and a Polychrome IV monochromator to maintain the excitation wavelength between 340 and 380 nm (TILL Photonics, Grafelfing, Germany). The cytosolic calcium concentration, total amount of calcium efflux from the ER (ER-releasable Ca^2+^), and calcium influx from the SOCE machinery were calculated using Origin 6.0 software (OriginLab, Northampton, MA, USA).

### Metaphase chromosome spread

The cells were treated with demecolcine (50 ng/ml, Sigma) overnight; the mitotic cells were subsequently collected by mitotic shake-off. The cells were resuspended in 75 mM KCl and incubated at room temperature for 20 min. The cells were then resuspended in freshly prepared Carnoy's solution (ice-cold methanol:glacial acetic acid = 3:1) for 30 min on ice. For chromosome spread analysis, the mitotic cells in Carnoy's solution were dropped onto glass slides, air-dried, and stained with DAPI. Images were acquired using a Leica DMI6000 inverted microscope.

### Lentivirus production and infection

Lentiviruses encoding STIM1 or control luciferase small-hairpin RNAs (shRNA) were obtained from the TRC lentiviral shRNA library in the National RNAi Core Facility of Academia Sinica, Taiwan. The targeting sequences of specific shRNAs are as follows: STIM1-1 (TRCN-0000149588: 5′- CGATGA GATCAACCTTGCTAA-3′); STIM1-2 (TRCN-0000358 717: 5′- TGGTGGTGTCTATCGTTATTG-3′); STIM1- 3 (TRCN-0000180131: 5′- CCACTCACAGTGGTTCTGT TT-3′); and luciferase (TRCN000072246: 5′- CAAAT CACAGAATCGTCGTAT-3′). Lentivirus production was performed according to the guidelines of the National RNAi Core Facility of Academia Sinica. To efficiently deplete endogenous STIM1, a mixture of STIM1-1, STIM-2, and STIM-3 lentiviruses was used.

### Xenograft tumorigenesis

For tumor xenograft experiments, BALB/c nu/nu nude mice (6–8 weeks old) were purchased from National Laboratory Animal Center, Taiwan and housed in microisolator cages at a specific pathogen-free facility. PreS2-LHBS cells were infected with recombinant lentiviruses expressing control or STIM1 shRNA for 48 h before being trypsinized, washed, and resuspended in PBS. A total of 1 × 10^6^ cells in 100 μl (PBS: matrigel = 1:1) were subcutaneously injected into the nude mice. Tumor volumes and numbers were measured at 2 months after injection. The expression of preS2-LHBS in tumor nodules was further confirmed by Western blotting.

## SUPPLEMENTARY MATERIALS FIGURES






