# Supplementary material for: Hepatitis B virus PreS2-mutant large surface antigen activates store-operated calcium entry and promotes chromosome instability
Source: Oncotarget. 2016 Mar 16;7(17):23346–60. doi: 10.18632/oncotarget.8109 (PMC5029631; doi:10.18632/oncotarget.8109)
Supplement: Supplementary file 1 [file oncotarget-07-23346-s001.pdf]

Hepatitis B virus PreS2-mutant large surface antigen activates store-operated calcium entry and promotes chromosome instability

Supplementary Materials

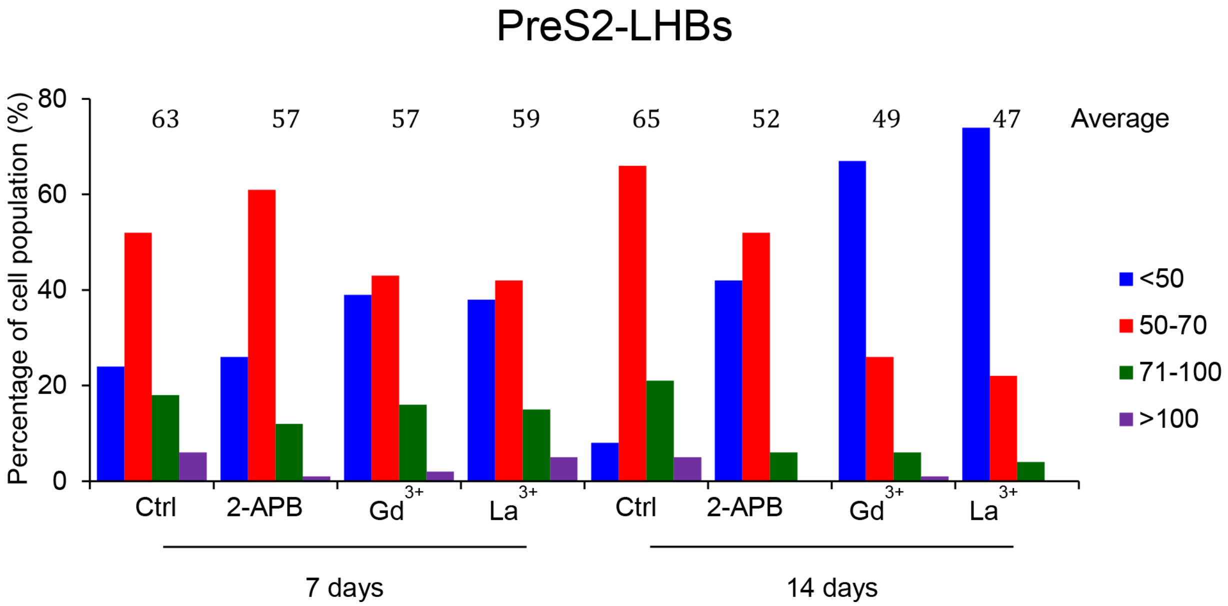

Supplementary Figure S1: SOCE inhibition reduced overall chromosome numbers in preS2-LHBs cells. Cells were treated with various SOCE inhibitors for 7 or 14 days before being analyzed by chromosome spreading. At least 200 chromosome spreads were counted in each group. Percentages of cells with different numbers of chromosomes are shown. Numbers shown on the top indicate average chromosome numbers of each treatment.

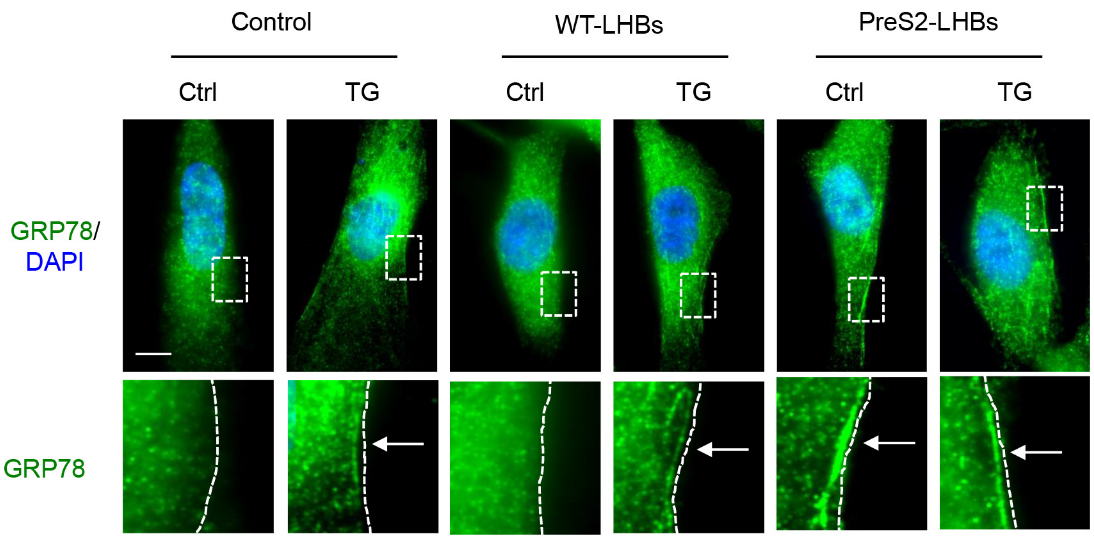

Supplementary Figure S2: Subcellular localization of GRP78 in response to ER stress. Control, WT-LHBs, and preS2-LHBs cells were treated with DMSO (ctrl) or 2 μM thapsigargin (TG) for 30 min and then fixed and stained with GRP78 (green) and DAPI (blue). The scale bar indicated 10 μm. The lower panel represents magnified images extracted from boxed areas of the upper panel. Peripheral recruitment of GRP78 (arrow-indicated, lower panel) was detected in control and WT-LHBs cells after TG treatment. In contrast, marginal GRP78 was detected in preS2-LHBs in the absence of TG. Dashed lines in the lower panel indicate the edge of the cell.

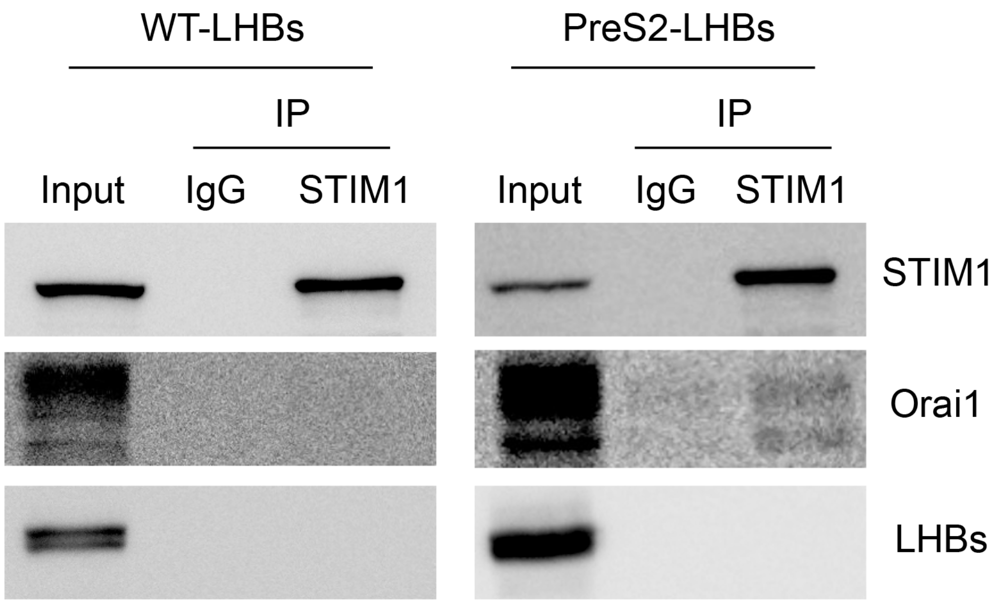

Supplementary Figure S3: STIM1 interacts with Orai1 in preS2-LHBs cells. Protein immunoprecipitations were performed with total cell lysates from WT-LHBs or preS2-LHBs cells, mouse IgG, and mouse anti-STIM1 antibody (BD, 610954). Immunoprecipitates were probed with mouse anti-STIM1, mouse anti-Orai1 (Santa Cruz, sc-377281), and mouse anti-preS1 (LHBs, clone 7H11). Neither WT-LHBs nor preS2-LHBs were precipitated together with STIM1. Orai1 was only pulled-down with STIM1 in preS2-LHBs cells, indicating the activation of SOCE in preS2-LHBs cells.

Supplementary Movies : Bipolar division. Differential interference contrast images of control cells were taken at 10-min interval for 150 minutes. Arrowheads indicate the cell underwent bipolar division and the two daughter cells. **Multipolar division.** Differential interference contrast images of preS2-LHBS expressing cells were taken at 10-min interval for 150 minutes. Arrowheads indicate the cell underwent multipolar division and the resulted three daughter cells.
